# The identification and characterisation of autophagy inhibitors from the published kinase inhibitor sets

**DOI:** 10.1042/BCJ20190846

**Published:** 2020-02-27

**Authors:** Maria Zachari, Julie M. Rainard, George C. Pandarakalam, Lindsay Robinson, Jonathan Gillespie, Muralikrishnan Rajamanickam, Veronique Hamon, Angus Morrison, Ian G. Ganley, Stuart P. McElroy

**Affiliations:** 1 Medical Research Council Protein Phosphorylation and Ubiquitylation Unit, School of Life Sciences, University of Dundee, Dow Street, Dundee DD1 5EH, U.K.; 2 European Screening Centre, University of Dundee, Biocity, Bo'Ness Road, Motherwell ML1 5UH, U.K.; 3 BioAscent Discovery Ltd, Biocity, Bo'Ness Road, Motherwell ML1 5UH, U.K.

**Keywords:** autophagy, chemical biology, high-throughput screening, kinases

## Abstract

Autophagy is a critical cellular homeostatic mechanism, the dysfunction of which has been linked to a wide variety of disease states. It is regulated through the activity of specific kinases, in particular Unc-51 like autophagy activating kinase 1 (ULK1) and Phosphatidylinositol 3-kinase vacuolar protein sorting 34 (VPS34), which have both been suggested as potential targets for drug development. To identify new chemical compounds that might provide useful chemical tools or act as starting points for drug development, we screened each protein against the Published Kinase Inhibitor Set (PKIS), a library of known kinase inhibitors. *In vitro* screening and analysis of the published selectivity profiles of the hits informed the selection of three relatively potent ATP-competitive inhibitors against each target that presented the least number of off-target kinases in common. Cellular assays confirmed potent inhibition of autophagy in response to two of the ULK1 inhibitors and all three of the VPS34 inhibitors. These compounds represent not only a new resource for the study of autophagy but also potential chemical starting points for the validation or invalidation of these two centrally important autophagy kinases in disease models.

## Introduction

Macroautophagy (referred to as autophagy here for simplicity) is a lysosome-dependent degradative pathway that recycles intracellular components such as proteins and/or whole organelles. Autophagy is a major homeostatic mechanism and can be activated as a survival response to various stresses including amino acid starvation and hypoxia. As such, dysregulated autophagy has been linked to numerous human pathologies ranging from cancer, diabetes and heart disease to the neurodegenerative disorders Parkinson's and Alzheimer's [1,2]. In the case of cancer particularly, increased autophagy levels have been linked to resistance to chemotherapy and radiation therapy. Given this, small molecule targeting of autophagy has been proposed to have therapeutic potential [3,4].

Autophagy is a multistage process that involves the *de novo* formation of a double membrane autophagosome: the organelle that surrounds and engulfs cytosolic components and shuttles them to the lysosomes for turnover and recycling. Initiation of autophagosome formation is regulated by the ULK1/2 complex (hereafter ULK1 complex), which contains the serine/threonine protein kinase ULK1 (or the very closely related ULK2) and its essential protein partners FIP200, ATG13 and ATG101 [5–7]. ULK1 kinase activation is crucial for autophagy initiation and its inhibition abolishes autophagy [8]. Upon activation the ULK1 complex in turn regulates the phosphorylation and activation of another kinase complex containing VPS34, the class III phosphatidylinositol 3-kinase, and its binding partners VPS15, BECLIN1 and ATG14L [9]. As with the ULK1 complex, the VPS34 complex is also essential for autophagy initiation. VPS34 catalyses the formation of the lipid phosphatidylinositol 3-phosphate (PI3P) at the site of autophagosome formation, which regulates the recruitment of PI3P-binding proteins such as WIPI2 and DFCP1 to aid in the expansion of the growing autophagosome [10].

To date, a limited number of small molecule inhibitors of ULK1 [11–13] and VPS34 [14–16] have been identified, which together provide very useful tools for studying the physiological and pathophysiological roles of these kinases. The complexity of the pathway and the tendency for kinase inhibitors to show broad spectrum kinase selectivity, however, means that further research would significantly benefit the field, from having access to a wider suite of high affinity, cell permeant chemical tools with well-understood profiles of pan-kinase selectivity. In an effort to promote research on functionally uncharacterised kinases and to encourage the sharing of data, GlaxoSmithKline (GSK) have made available two Published Kinase Inhibitor Sets (PKIS) that together constitute a library of 856 compounds, which were formerly part of GSK's kinase inhibitor research programmes [17–19]. This library provides a diversity of chemical scaffolds, comprising mainly of kinase hinge binding motifs, which have been screened for inhibition against a very wide range of kinases. All these kinase inhibition data are freely available through the chemical database of bioactive molecules with drug-like properties (ChEMBL) and provide a significant resource for discovering new chemical probes against relatively understudied kinases. Here we describe the output from two screens of the PKIS against recombinant ULK1 and VPS34, which, following assessment of the published selectivity profiles, resulted in the identification of two new ULK1 inhibitors and three new VPS34 inhibitors that exhibit good efficacy in cellular assays of autophagy.

## Experimental procedures

### Reagents

Original samples from the PKIS were obtained from GSK. The compounds GW301784X, GW837331X, GW406108X, GSK2358994A, GSK846226A and GW429374A were synthesised according to previously published methods [20–25]. Protein kinases (ULK1, VPS34 and AMPK α1β2ɣ1) and SAMS peptide were purchased from MRC PPU Reagents and Services, Dundee, Scotland. ADP Hunter™ Plus kits and ultra-pure ATP Gold were from Discoverx. SBI-0206965 and VPS34-IN1 were obtained from Selleck Chemicals. MRT68921 was provided by LifeArc (formerly MRC-Technology). Trizma® hydrochloride, Trizma® base, Tris(2-carboxyethyl)phosphine (TCEP), Pluronic® F-127, sodium chloride (NaCl), magnesium chloride (MgCl_2_), manganese chloride (MnCl_2_), ethylene glycol-bis(β-aminoethyl ether)-N,N,N′,N′-tetraacetic acid (EGTA), dimethyl sulfoxide (DMSO) and bovine myelin basic protein (MBP) substrate were from Sigma–Aldrich. 4-(2-hydroxyethyl)-1-piperazineethanesulfonic acid (HEPES), Tween-20 and PI:PS lipid kinase substrate were from Thermo Fisher Scientific. Reagents used for Western blot and immunofluorescence were published previously [26].

### Antibodies

anti-pS757 ULK1, anti-pS555 ULK1 and anti-ULK1 were from CST, anti-pS318 ATG13 from Novus, anti-ATG13 from Sigma–Aldrich, anti-α-Tubulin from Proteintech and sheep anti-LC3b (S400D) from MRC PPU Reagents and Services. All antibodies were used for Western blotting at 1:1000 dilution apart from the LC3b antibody which was used at 1:500. HRP-conjugated secondary anti-sheep, anti-rabbit and anti-mouse antibodies were purchased from Thermo Scientific and used in 1:10 000 dilution. For immunofluorescence, anti-WIPI2 (Bio-Rad), anti-ULK1 (CST) and anti-LC3 (MBL) antibodies were used in 1:500 dilution. Secondary antibodies for immunofluorescence (anti-mouse Alexa Fluor® 488 and anti-rabbit Alexa Fluor® 594) (Thermo Scientific) were used in 1:1000 dilution.

### Kinase assays

The kinase activity assays for ULK1, VPS34 and AMPK are based on the ADP Hunter™ Plus technology developed by Discoverx [27]. This technology relies on a coupled assay system to measure the amount of ADP product formed during the kinase reaction. Each kinase activity assay was run as an endpoint assay. For this, a suitable reaction time was selected to ensure that ATP conversion was a linear function of time as it was well within the steady-state phase of the reaction. Additionally, each assay was run using an ATP concentration corresponding to the experimentally determined *K*_M (ATP)_ apparent in order to allow compound selectivity ranking between the kinases.

Kinase reactions for AMPK were performed in assay buffer containing 15 mM HEPES, 20 mM NaCl, 1 mM EGTA, 0.02% Tween 20, 10 mM MgCl_2_, 0.1% bovine gamma globulins, pH 7.4 (buffer included in the ADP Hunter™ Plus kit) with 20 µM ATP, 25 µM SAMS peptide substrate and 1 nM AMPK α1β2ɣ1 kinase. Kinase reactions for ULK1 were performed in assay buffer containing 50 mM Tris–HCl, 1 mM EGTA, 10 mM MgCl_2_, 0.02% Tween 20 pH 7.0 with 60 µM ATP, 20 µM MBP substrate and 20 nM ULK1 kinase. Kinase reactions for VPS34 were performed in assay buffer containing 50 mM HEPES, 2 mM TCEP, 0.1% Pluronic F-127, 25 mM NaCl, 5 mM MnCl_2_, pH 7.0 with 50 µM ATP, 10 µM PI:PS lipid kinase substrate and 80 nM kinase. The kinase reactions were run for 45 min (VPS34), 60 min (AMPK) or 4 h (ULK1) at room temperature before being stopped by addition of detection reagents and further incubation for 15–60 min at room temperature. The fluorescent intensity signal was measured using a PHERAstar microplate reader (BMGLabtech) using excitation / emission wavelengths of 530/590 nm. In inhibitor screening experiments on the original PKIS samples, the final concentration of DMSO was 0.25% DMSO. For *K*_M_ determination and mechanism of inhibition experiments, kinase assays were run using the conditions described previously except that the ATP or substrate concentrations were varied, and the reaction time was adjusted to ensure that each was monitored during linear, steady-state conditions. For compound testing, the final concentration of DMSO in the assay was between 0.25–0.99% DMSO. Orthogonal kinase assays using radiolabelled ATP for ULK1, ULK2 and AMPK were performed by MRC PPU Reagents and Services as described previously [28].

### Cell culture and treatments

U2OS (human osteosarcoma-derived) cells were cultured in DMEM supplemented with 10% foetal bovine serum, 100 U/ml Penicillin and 2 mg/ml l-glutamine. Cells were pre-treated with compounds at the indicated concentrations for 60 min followed by treatments with EBSS or EBSS combined with/without 50 nM Bafilomycin A1 (Enzo Life Sciences) for a further 60 or 120 min with/without inhibitors, as indicated in figures/figure legends.

### Western blotting

Prior to western blot analysis, cells were washed twice in ice-cold PBS and lysed in buffer containing 50 mM HEPES pH 7.4, 150 mM NaCl, 1 mM EDTA, 10% Glycerol, 0.5% NP-40, 1 mM DTT, supplemented with phosphatase inhibitors (1.15 mM sodium molybdate, 4 mM sodium tartrate, 0.01 M β-glycerophosphate, 1 mM sodium fluoride, 1 mM sodium orthovanadate) and protease inhibitor tablets (Roche) according to manufacturer's instructions. Lysates were then spun at 20 000×***g*** for 10 min and the supernatant was collected for protein content estimation using Protein Assay Dye Reagent Concentrate from Bio-Rad. 4× LDS was added to the lysate (final dilution 1×) prior to heating at 95°C for 5 min. Electrophoresis and transfer to PVDF membranes was performed using standard protocols. Membranes were blocked in 5% milk in TBS-T prior to incubation with primary antibodies (in 5% milk/TBS-T for ULK1, pS555 (human S556) ULK1, pS757 (human S758) ULK1, pS318-ATG13, ATG13, and in 5% BSA/TBS-T for LC3 and α-Tubulin) under rotation overnight at 4°C followed by washes in TBS-T and incubation with HRP-conjugated secondary antibodies (in 5% milk in TBS-T) for 60 min at room temperature. Membranes were washed in TBS-T before application of ECL reagent (GE healthcare) and development on hypersensitive X-ray films (GE healthcare) (Figures 5A, 6C and Supplementary Figure S7C) or using BIO-RAD Clarity^TM^ western blotting substrate (Supplementary Figure S6C and Supplementary Figure S7A) and a BIO-RAD ChemiDoc^TM^ imaging system.

### Immunofluorescence and imaging

Prior to immunofluorescence, cells were grown on glass coverslips to ∼60% confluency. Cells were washed in PBS prior to fixation with 3.7% PFA/10 mM HEPES pH 7.0 at room temperature for 20 min. PFA was quenched using DMEM/10 mM HEPES pH 7.0/0.02% NaN_3_, followed by permeabilisation with 0.2% NP-40 in PBS for 5 min, followed by blocking with 1% BSA/PBS/0.02% NaN_3_. Primary antibody incubation was at room temperature for 60 min, followed by two 10-minute washes, then fluorescent secondary antibody incubation for 30 min and further washes (all in blocking buffer) before mounting onto glass slides. A wide field Nikon TS microscope was used for image capturing.

### Quantification and statistical analysis

Quantification of enzyme kinetics was accomplished by calculating the linear, steady-state reaction velocities at varying substrate concentrations and fitting to the Henri–Michaelis–Menten equation using GraphPad Prism to derive *K*_M_ and *V*_max_. Inhibitor activity was normalised to positive and negative controls to yield %Inhibition:

$$\text{\%}\text{Inhibition}=100\;\times \;\frac{\text{Sample}-{\tilde{\text{X}}}_{\mathrm{Max}}}{{\tilde{\text{X}}}_{\mathrm{Min}}-{\tilde{\text{X}}}_{\mathrm{Max}}}$$

$\tilde{X}$*_Max_* = Median fluorescence for the maximum inhibition controls (enzyme plus maximal effective concentration of reference inhibitor − 100% control)

$\tilde{X}$*_Min_* = Median fluorescence for the no inhibition controls (enzyme plus vehicle − 0%)

Inhibitor potency was determined by fitting %inhibition data at varying inhibitor concentrations to a four-parameter logistic equation:

$$\text{y}=\text{A}+\;\frac{\left(\text{B}-\text{A}\right)}{1+{\left(\frac{x}{\text{I}{\text{C}}_{50}}\right)}^{n}}$$

A = lower asymptote (% inhibition in the absence of compound ∼0%)

B = upper asymptote (% inhibition at ≥maximally effective concentration of compound ∼100%)

IC_50_ = the half maximal inhibitory concentration

*n* = Hill's slope of the curve (related to the steepness of the curve at the IC_50_).

Quantification of western blots was performed with band densitometry analysis using ImageJ. Quantification of LC3 puncta was performed using either the ITCN plugging in ImageJ or Volocity. One-way or two-way Anova (using GraphPad Prism) followed by a post-hoc test, was used for statistical analysis of western blots and immunofluorescence as indicated in figure legends.

### Compound docking

The Schrödinger Suite (Schrödinger, New York) was used to prepare the structures (PrepWizard) and to perform the docking experiments (Glide). The docking was run using the Extra Precision (XP) method and the parameters were assigned to their default values. Several co-crystallised structures were available in the Protein Data Bank for both kinases and in each set the DFG-in conformations were very similar. 4WNO and 4UWF entries were selected for ULK1 and VPS34, respectively.

## Results

### Screening of PKIS against ULK1 and VPS34

**Figure 1.**
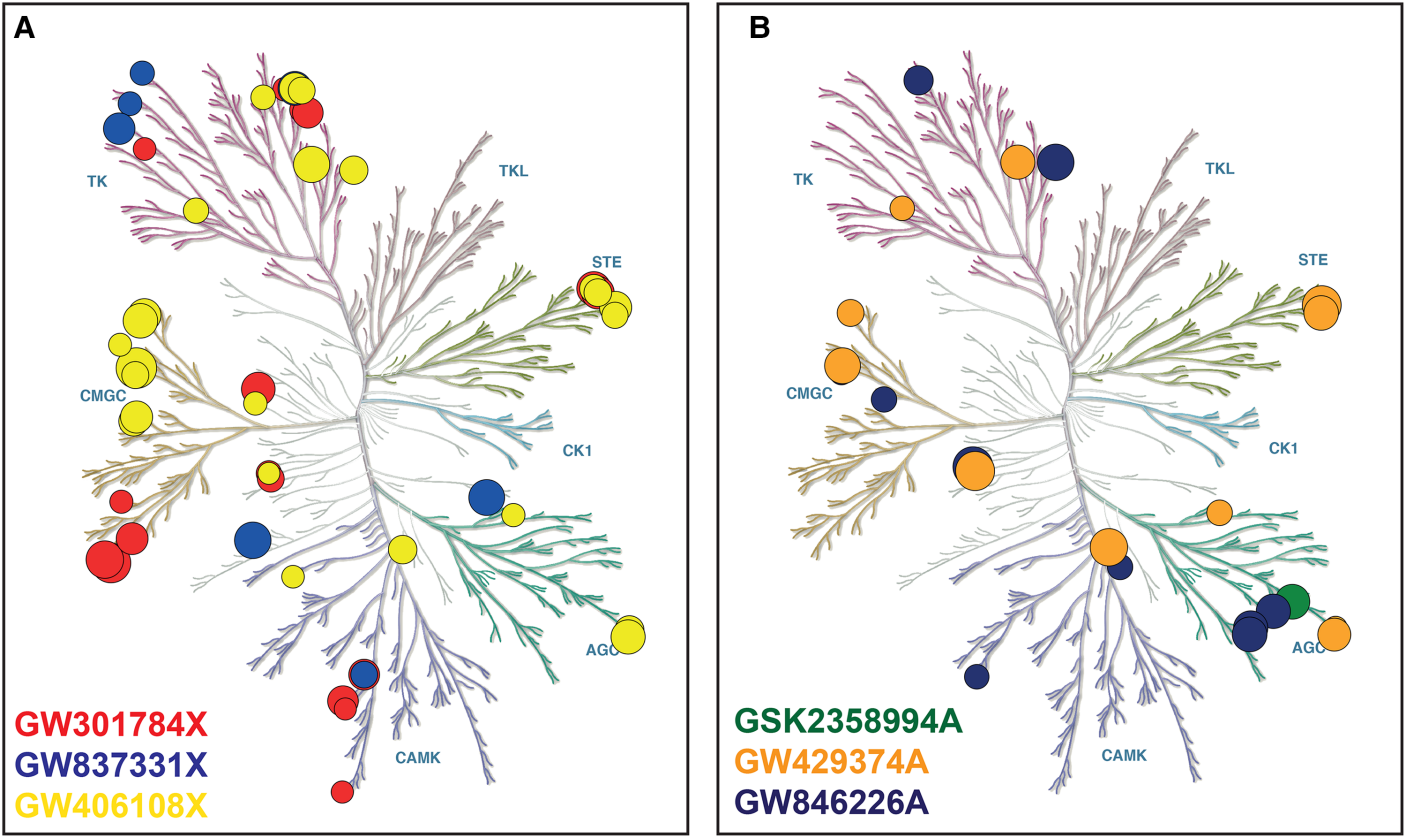
Kinome selectivity profiles for the three ULK1 and three VPS34 inhibitors identified in the screen. Kinase inhibition data extracted from the ChEMBL database (https://www.ebi.ac.uk/chembl/) for (**A**) the ULK1 hits and (**B**) the VPS34 hits. The figures were generated using Kinmap beta (www.kinhub.org/kinmap/) [43] and illustration of the human kinome phylogenetic tree represents courtesy of Cell Signalling Technology (CST) (www.cellsignal.com). The size of the circles represents % inhibition, with the smallest circle representing inhibition = 50%. Each coloured branch of the phylogenetic tree represents a different kinase group abbreviated as follows: TK, Tyrosine Kinases; TKL, Tyrosine Kinase-Like; STE, Homologues of the yeast STE7, STE11 and STE20 genes; CK1, casein kinases; AGC, the protein kinase A, G, and C families; CAMK, modulated by Calcium/Calmodulin; CMGC, cyclin-dependent kinases (CDKs); MAP kinases, mitogen-activated protein kinases; GSK, glycogen synthase kinases and CDK-like kinases.

The PKIS library of 856 compounds was screened at 100 nM and 1 µM against ULK1 in the ADP Hunter Plus assay. Seventy-nine compounds showing ≥10% inhibition (corresponding to 5× the robust standard deviation from the median) at both concentrations were selected to be retested in a concentration-dependent format. The ULK1 potency data were then assessed in combination with the kinase selectivity data available in ChEMBL to identify three compounds with differing/complimentary selectivity profiles (GW301784X, GW837331X and GW406108X), which were then resynthesized. These compounds were relatively potent against ULK1 with pIC_50_s greater than 6 and they appear to inhibit a minimum number of off-target kinases in common with one another (Figure 1A and Supplementary Table S1). The resynthesized samples were confirmed inhibitors of ULK1 with pIC_50_ values of 7.28 ± 0.06 (53 nM), 6.19 ± 0.06 (646 nM) and 6.37 ± 0.02 (427 nM), respectively (Table 1). ULK1 inhibition was also confirmed in a standard ^32^P-ATP filter plate assay to confirm inhibition using an orthogonal method.

**Table 1:** The Structure of the selected hit compounds and their potency against ULK1, VPS34 and AMPK (mean pIC_50_ ± SD, *n* = 5, mean potency in nM in parenthesis)

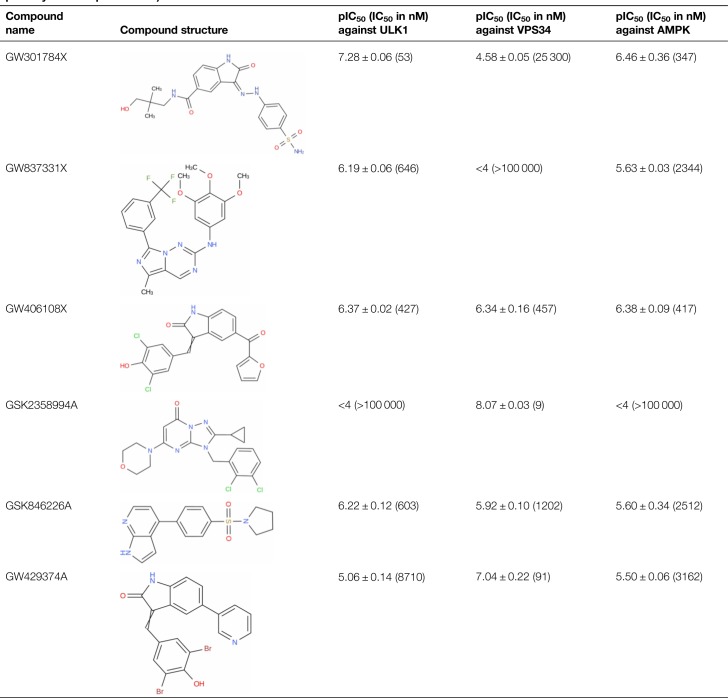

The PKIS library was also screened at 100 nM and 1 µM against VPS34 in the ADP Hunter Plus assay. 104 hits showing ≥20% inhibition at 100 nM were selected to be retested in a concentration-dependent format. As with the follow up of the ULK1 screen, the pan-kinase selectivity data available in ChEMBL were assessed for all the hits and three compounds with complimentary selectivity profiles (GSK2358994A, GSK846226A and GW429374A) were resynthesized. These compounds had pIC_50_ values above 6 against VPS34 and the least number of off-target kinases in common with one another (Figure 1B and Supplementary Table S1). Inhibition of VPS34 was confirmed for the resynthesized hits with pIC_50_ values of 8.07 ± 0.03 (8.6 nM), 5.92 ± 0.1 (1.2 µM) and 7.04 ± 0.22 (90.5 nM), respectively (Table 1).

With exception of GSK2358994A, all resynthesized ULK1 and VPS34 hits were weakly active against AMPK α1β2ɣ1 kinase in the ADP Hunter Plus assay with pIC_50_ values ranging from 5.5 to 6.5 which was confirmed in the ^32^P-ATP radioactive orthogonal assay (Table 1). All 6 compounds were also tested in a ULK2 radiometric assay and showed similar inhibition to that observed at ULK1 against this highly related kinase (data not shown).

### Mechanism of inhibition

**Figure 2.**
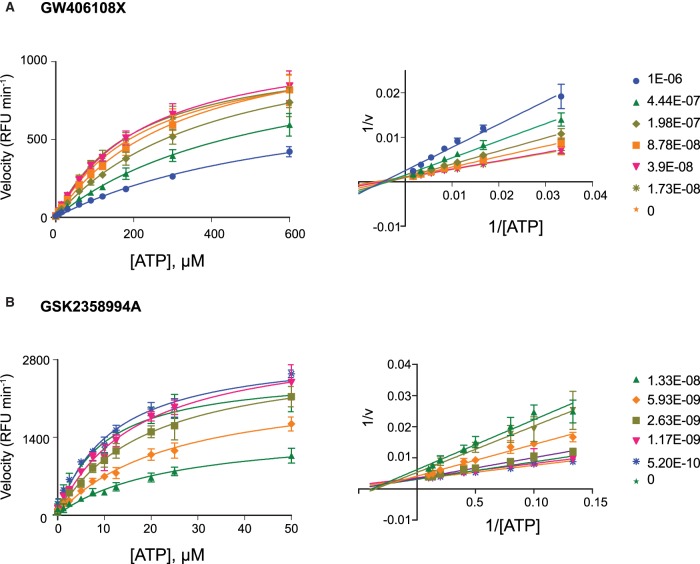
GW406108X and GSK2358994A primarily exhibit ATP competitive inhibition against their respective targets. Michaelis–Menten and Lineweaver–Burke plots showing inhibition of (**A**) ULK1 by GW406108X and (**B**) VPS34 by GSK2358994A at varying concentrations of ATP. The data show the average of three independent experiments; error bars indicate the standard deviation.

Depending upon the primary target they were identified to inhibit, the resynthesized hits were tested against either ULK1 or VPS34 at varying substrate concentrations. The testable range of ATP concentrations were restricted to below 600 µM for ULK1 and below 200 µM for VPS34 due to substrate inhibition. This was also the case for the VPS34 lipid substrate PI:PS which was restricted to below 12 µM. Michaelis–Menten plots and Lineweaver–Burk double reciprocal linear representation show that GW301784X, GW837331X and GW406108X display a profile indicating primarily ATP competitive inhibition against ULK1 [29] (Figure 2A and Supplementary Figure S1) and clear non-competitive inhibition with respect to MBP (Supplementary Figure S2). Similarly, GSK2358994A, GSK846226A and GW429374A display a profile indicating primarily ATP competitive inhibition against VPS34 (Figure 2B and Supplementary Figure S3) and clear non-competitive inhibition with respect to PI:PS (Supplementary Figure S4).

### Modelling the compound-target binding mode

A structural comparison of the ULK1 and VPS34 binding sites show major differences in shape, correlated with important variations in the backbone topology and in the nature and size of some of the residues lining it. A straightforward rationale regarding the selectivity of the compounds against their target was thus difficult to infer from the binding site analysis.

**Figure 3.**
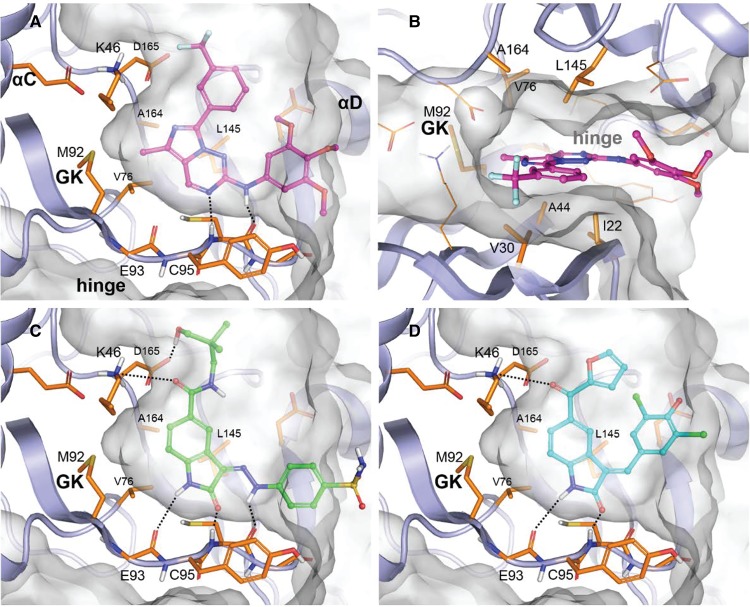
Docking of ULK1 inhibitors. Docking poses of ULK1 inhibitors, compound GW837331X in (**A**) lateral view and (**B**) top view, (**C**) compound GW301784X and compound (**D**) GW406108X.

**Figure 4.**
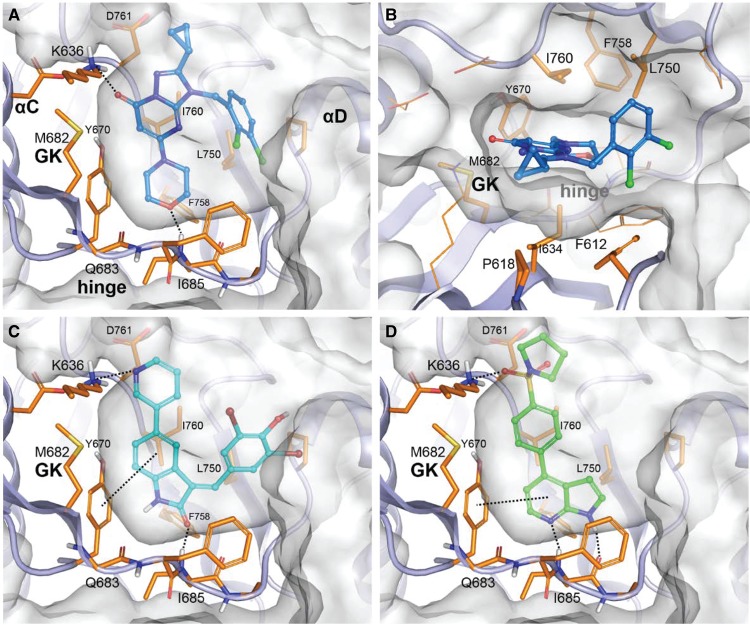
Docking of VPS34 inhibitors. Docking poses of VPS34 inhibitors, compound GSK2358994A in (**A**) lateral view and (**B**) top view, (**C**) compound GW429374A and (**D**) compound GSK846226A.

The docking pose suggested for each compound into its target is depicted in Figure 3 for ULK1 and Figure 4 for VPS34. The proposed binding modes to the hinge were observed in PDB structures with compounds similar to our set and co-crystallised in various kinases targets. For clarity, some sidechains and/or backbone regions are not represented. In both kinases the core of the compounds are clamped in the hydrophobic centre of the pocket formed by aliphatic and/or aromatic residues on the N lobe (Ile22, Val30, Ala44 in ULK1, Phe612, Pro618, Ile634 in VPS34) and the C lobe (Val76, Leu145, Ala164 in ULK1, Tyr670, Leu750, Ile760, Phe758 in VPS34) (shown in panel b in each figure). Regarding the ULK1 inhibitors, for GW837331X the hinge binding is mediated through a nitrogen of the imidazotriazine core and the trimethoxyaniline nitrogen oriented toward the crevice entrance (Figure 3A,B). A methyl substituent coming off the imidazole ring and pointing in the direction of the gatekeeper improves the occupancy of this buried region by the compound. Both GW301784X (Figure 3C) and GW406108X (Figure 3D), present an indolin-2-one as a common core establishing two hydrogen-bond interactions with the hinge through the nitrogen and the carbonyl oxygen contacting the backbone carbonyl of Glu93 and the backbone amide of Cys95, respectively. In the case of GW301784X, an extra hydrogen-bond interaction is present between the aniline nitrogen and the backbone carbonyl of Cys95. Opposite from the indolin-2-one carbonyl position, both compounds contain a carbonyl group that is hydrogen bound to the sidechain amine of Lys46 above the gatekeeper.

Regarding the VPS34 inhibitors, GSK2358994A is similar to a series of selective VPS34 inhibitors identified by Pasquier et al. [30] in which the hinge binding motif is a morpholine group (Figure 4A,B). The superimposition of GSK2358994A docked into 4UWF with the ligand extracted from this structure shows a very good shape overlap (Supplementary Figure S5). In both cases, two main hydrogen bonds stabilise the compound, the morpholine oxygen interacting with the hinge backbone nitrogen of Ile685 and the pyrimidinone carbonyl with Lys636 amine (via a conserved water molecule in the crystal). Although not clearly suggested by the docking, the cyclopropyl could fit into a hydrophobic sub-pocket in a similar manner to the trifluoromethyl of 4UWF ligand as described by the authors for this structure. In GW429374A (Figure 4C), the hinge binding is mediated through the indolin-2-one carbonyl and Ile685 backbone nitrogen and potentially through a second one between its nitrogen and Gln683 backbone carbonyl (not clearly suggested by the docking in this conformation of the binding site). GSK846226A links to the hinge via both nitrogen atoms of its pyrrolopyridine interacting with Ile685 backbone moiety. Both compounds are hydrogen bonded to Lys636 amine, through a pyrimidine in GW429374A or a sulfonyl carbonyl in GSK846226A, and an extra stabilisation is added by their aromatic hinge binder moiety that forms an edge-to-face Pi-Pi interaction with Tyr670.

### GW837331X and GW406108X inhibit cellular ULK1 kinase activity and block autophagic flux

**Figure 5.**
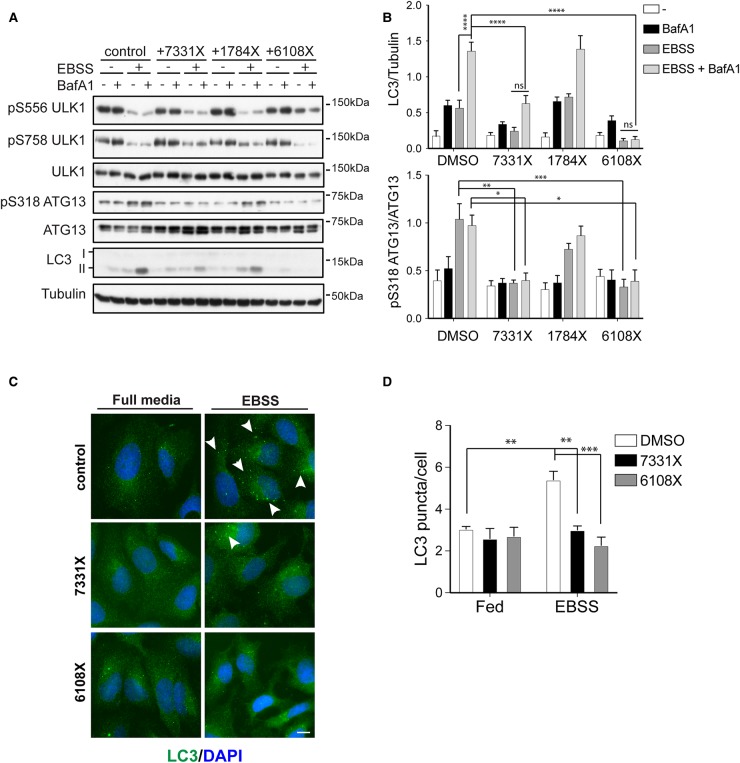
GW837331X and GW406108X, but not GW301784X inhibit ULK1 and autophagy flux in cells. (**A**) U2OS cells were pre-treated for 60 min with or without 5 µM of each inhibitor as indicated following by 60 min treatment with or without EBSS in the presence or absence of 50 nM BafA1. Cells were subsequently subjected to western blot analysis with antibodies against the indicated proteins. (**B**) Quantification of pS318 ATG13 normalised to total ATG13 and LC3 normalised to GAPDH. Graph bars represent mean from *n* = 5 experiments ± SEM and statistics were carried out using two-way ANOVA and Bonferroni's post-hoc test. (**C**) As in (**a**) but upon treatments cells were fixed and stained for endogenous LC3 (in green). Arrowheads indicate LC3 puncta appearing upon EBSS treatment. Scale bar 10 µm. (**D**) Quantification of LC3 puncta using the ITCN plugin in ImageJ. Graph bars represent mean from *n* = 3 experiments, error bars ± SEM and statistics were carried out using two-way ANOVA and Bonferroni's post-hoc test. * *P* ≤ 0.05, ** *P* ≤ 0.01, *** *P* ≤ 0.001, **** *P* ≤ 0.0001 and ns = non-significant.

ULK1 kinase activity can be studied in cells by monitoring phosphorylation of serine 318 (pS318) of ATG13, a well-established substrate of ULK1 [11,31]. Amino acid starvation with EBSS, results in increased ULK1 activity as observed by the two-fold increase in pS318 ATG13 levels (Figure 5A,B). In the presence of 5 µΜ GW837331X or GW406108X, the starvation-induced increase in ATG13 phosphorylation was significantly reduced (Figure 5A,B). In contrast, GW301784X did not have a significant effect on pS318 ATG13 in cells, despite showing activity against ULK1 *in vitro*. To monitor autophagic flux, the levels of the autophagosomal marker protein LC3 were measured in the presence and absence of the vacuolar ATPase inhibitor Bafilomycin A1 (BafA1). An increase in autophagic flux is evidenced by an increase in the lipidated form of LC3 (LC3-II) upon BafA1 treatment [32]. As expected, amino acid starvation resulted in increased LC3 flux and in-line with their ability to inhibit ULK1, both GW837331X and GW406108X significantly blocked this (Figure 5A,B). Consistent with its lack of ULK1 inhibition in cells, GW301784X did not block LC3 flux. GW837331X and GW406108X were acting upon ULK1 and autophagy without affecting mTOR or AMPK activity as shown by monitoring ULK1 pS758 (mTOR site) or pS556 (AMPK site) levels. To further confirm these results, we performed imaging experiments monitoring endogenous LC3 levels in U2OS cells. As shown in Figure 5C, LC3 puncta formation increases upon EBSS treatment and this increase is abolished when EBSS is combined with GW837331X or GW406108X (Figure 5C,D). Notably GW837331X and GW406108X, did not appear to cause the formation of enlarged and stalled autophagosomes, as has been reported for MRT68921 [11]. To analyse this in more detail, we directly compared LC3 puncta formation following amino acid starvation in cells treated with GW837331X, GW406108X and MRT68921 (Supplementary Figure S6). We also included the previously published and structurally distinct ULK1 inhibitor SBI0206965 as an additional control [12]. Both MRT68921 and SBI0206965 treatment resulted in LC3 puncta that were larger than those formed with EBSS alone, implying that the stalled autophagosome phenotype is not a unique feature of MRT68921, but is indeed related to ULK1 inhibition (Supplementary Figure S6A,B). However, consistent with earlier results, GW837331X and GW406108X did not show this phenotype. We do not yet fully understand these differences, but it is worth mentioning that both MRT68921 and SBI0206965 inhibited ULK1 more potently compared with the ULK1 inhibitors identified here (Supplementary Figure S6C) and are more specific, which could explain these phenotypic differences. Regardless, taken together our data show that two of the three *in vitro* ULK1 inhibitors we shortlisted significantly reduce cellular ULK1 activity and autophagic flux induced by amino acid starvation.

### GSK2358994A and GW429374A inhibit cellular VPS34 activity and block autophagic flux

**Figure 6.**
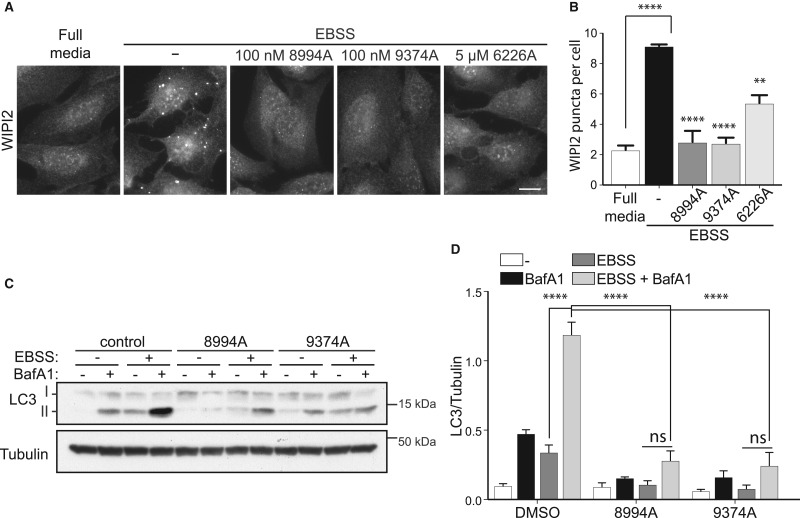
GSK2358994A and GW429374A potently inhibit VPS34 and autophagy flux in cells, whereas GW846226A inhibits VPS34 less potently. (**A**) U2OS cells were pre-treated for 60 min with or without 100 nM of each inhibitor as indicated following by 60 min treatment with or without EBSS. Cells were then fixed and stained for WIPI2. Scale bar 10 µm. (**B**) Quantification of WIPI2 puncta using the ITCN plugin in ImageJ. (**C**) As in (**A**) but upon treatments cells were subjected to western blot analysis with antibodies against the indicated proteins. (**D**) Quantification of LC3 normalised to Tubulin. Graph bars represent mean from *n* = 3 experiments, error bars ± SEM and statistics were carried out using two-way ANOVA and Bonferroni's post-hoc test. ** *P* ≤ 0.01, **** *P* ≤ 0.0001.

Given the evidence that GSK2358994A and GW429374A are potent and selective VPS34 inhibitors, we next tested their effects on VPS34 lipid kinase activity and autophagy induction in cells. VPS34 activity is indispensable during autophagosome biogenesis by producing PI3P, which acts as a signalling molecule for the recruitment of downstream PI3P-binding autophagy proteins such as DFCP1 and WIPI2 [33,34]. As previously reported, loss of PI3P results in dramatic inhibition of autophagy in cells [10]. Given that PI3P is necessary to recruit WIPI2 to initiating autophagosomes, autophagy-specific PI3P production can be monitored indirectly by measuring WIPI2 puncta formation, which significantly increase during EBSS-induced autophagy. When cells were treated with GSK2358994A or GW429374A, in combination with EBSS treatment, WIPI2 puncta were significantly reduced compared with the EBSS only treated samples (Figure 6A,B). This strongly suggests that VPS34 activity is indeed inhibited and that both GSK2358994A and GW429374A are potent inhibitors of VPS34, working at concentrations as low as 100 nM in cells (Figure 6A,B). As with the ULK1 inhibitors, we performed LC3 flux assays under EBSS starvation conditions and observed that both GSK2358994A and GW429374A significantly reduced LC3 flux as expected (Figure 6C,D). Furthermore, we monitored the activity of the upstream kinases mTOR, AMPK and ULK1, upon treatments with GSK2358994A and GW429374A. Monitoring phosphorylation of pS758 ULK1 and pS556 ULK1 showed that mTORC1 and AMPK kinases respectively, were unaffected upon treatment with the compounds (as well as upon treatment with the recently published selective VPS34 inhibitor, VPS34-IN1 [14]). Thus supporting that autophagy flux inhibition is due to a direct block in VPS34 activity rather than a change in upstream signalling (Supplementary Figure S7A,B). ULK1 activity was still increased upon EBSS treatment in the presence of VPS34 inhibitors, although not significantly (Supplementary Figure S7A,B). It is possible that in cells ULK1 activity is marginally affected by the presence of VPS34 inhibitors, potentially due to a negative feedback loop, as these inhibitors were not potent against ULK1 *in vitro* (Table 1). Given that the GSK2358994A is a potent and highly specific kinase inhibitor (Figure 1B), we decided to compare its ability to block LC3 flux with that of VPS34-IN1 [14]. Both inhibitors similarly blocked EBSS-induced autophagy flux at concentration as low as 10 nM, with a greater effect at 100 nM (Supplementary Figure S7C). GSK846226A was also tested but was less effective with an inhibitory effect at 5 µM (and not lower concentrations) in reducing WIPI2 puncta formation (Figure 6A,B). It is possible that the cellular uptake of GSK846226A is lower compared with the other VPS34 inhibitors or, perhaps more likely (given the observations with recombinant protein — Table 1) that it is not as potent a VPS34 inhibitor (Figure 6A,B).

## Discussion

Given the significant research focus on the molecular events governing autophagy and the potential role it plays in disease processes, there is a requirement for new, potent and selective tool inhibitors. A historically bountiful source of druggable targets have been the protein kinases, which have been a major focus of drug development efforts in the pharmaceutical industry [35]. A major research output of this effort has been the development of the PKIS, which is freely available to researchers to use as a focussed compound library for identifying tool compounds for new and emerging kinases [17–19]. We therefore decided to use the PKIS to screen for inhibitors of the two main autophagy kinases, ULK1 and VPS34.

ULK1 is the earliest autophagy-specific target during autophagosome biogenesis and autophagy initiation. The role of ULK1 seems to be primarily for autophagy initiation (although reports have suggested potential autophagy-independent roles) which makes it ideal as a target to inhibit autophagy [8,36]. Recently, a small number of compounds that inhibit ULK1 kinase activity have been published: Compound 6 [37,38], MRT68921 [11], SBI0206965 [12], and ULK1-101 [13]. However, only the latter three were shown to block autophagy in cellular assays, with MRT68921 being the most potent, although SBI0206965 and ULK1-101 were shown to have improved selectivity profiles. Here, we have identified two compounds, GW837331X and GW406108X, that inhibit recombinant ULK1 kinase activity and block autophagy in cells, without affecting the upstream signalling kinases mTORC1 and AMPK. Though these inhibitors potently block ULK1 activity and autophagy, the data from ChEMBL indicate that they also inhibit quite a range of additional kinases and are likely, therefore, to exhibit multiple off-target effects. That said, their unique chemical structures when compared with the previously published ULK1 inhibitors and the lack of off-target kinases in common suggests they offer use as chemical tools in the study of the cellular function of ULK1. Interestingly the most potent inhibitor of recombinant ULK1 that we identified, GW301784X, did not show inhibition of cellular ULK1. This highlights one of the complexities of drug discovery and development whereby activity in simpler model systems does not always translate into more a complex environment. This may reflect genuine differences in the target pharmacology between recombinant vs natively expressed ULK1 but more likely indicates difficulties in obtaining high enough intracellular compound concentrations. This can be due to factors such as low membrane permeability, high non-specific binding to off-target proteins in the growth media and/or intracellularly, low compound stability in media, the activity of cellular catabolic enzymes and the action of cellular transporters/efflux pumps. As the PKIS is a library of compounds which predominantly target the ATP binding site, it was reasonable to use this as the hypothesis for modelling the binding mode of the hits. Indeed, the structures of the compounds resemble compound-kinase structures published in the PDB and the mechanism of inhibition experiments provide supporting evidence for ATP competition.

In our effort to discover more selective and potent autophagy inhibitors, we screened the same library against the second autophagy-essential kinase, the class III phosphatidylinositol-3 kinase VPS34. We found that two compounds, namely GSK2358994A and GW429374A, are very potent VPS34 inhibitors as they inhibit PI3P formation (by indirect measurement of the autophagy PI3P binding protein WIPI2) and concomitantly autophagy flux in cells. Of the two VPS34 inhibitors, GSK2358994A, displays a highly impressive selectivity for VPS34, although it is also potent against PI3Kδ, which is known to primarily express in leucocytes. Therefore, we propose that GSK2358994A will provide a very useful new tool in the study of VPS34 function in autophagy and other cellular processes. Many studies still utilise the amino acid analogue 3-methyladenine (3-MA) as a method to inhibit VPS34 and autophagy. This compound, first identified as an autophagy inhibitor by Seglen et al. [39] has been instrumental in many key mechanistic autophagy studies. However, its very low potency and lack of specificity means that it cannot be recommended for future work given the next generation of specific VPS34 inhibitors discussed here and elsewhere [14–16,30,40,41]. It should be noted that VPS34's cellular roles are not limited to autophagy as it also plays a critical role in endocytic trafficking [42]. Hence, the ability to target autophagy at multiple nodes not only offers a way to mechanistically decipher distinct stages of autophagy induction, but also offers a way to more potently block the pathway.

## Abbreviations

3-MA: 3-Methyladenine
ADP: adenosine diphosphate
AMPK: AMP-activated protein kinase
ATG: autophagy related
ATG14L: autophagy related 14-Like
ATP: adenosine triphosphate
BSA: bovine serum albumin
DFCP1: double FYVE-Containing Protein 1
DMSO: dimethyl sulfoxide
FIP200: FAK Family Kinase-Interacting Protein Of 200 KDa
GSK: GlaxoSmithKlein
HRP: horseradish peroxidase
LC3: microtubule-associated protein 1A/1B-light chain 3
LDS: lithium dodecyl sulfate
MBP: myelin basic protein
PBS: phosphate-buffered saline
PDB: Protein data bank
PFA: paraformaldehyde
PI: phosphatidylinositol
PI3P: Phosphatidylinositol 3-phosphate
PKIS: published kinase inhibitor set
PS: phosphatidylserine
TBS-T: Tris-buffered saline-Tween 20
ULK1/2: Unc-51 like autophagy activating kinase
VPS34: vacuolar protein sorting 34
WIPI2: WD repeat domain, phosphoinositide interacting 2
