# Supplementary material for: The identification and characterisation of autophagy inhibitors from the published kinase inhibitor sets
Source: Biochem J. 2020 Feb 27;477(4):801–14. doi: 10.1042/BCJ20190846 (PMC7054748; doi:10.1042/BCJ20190846)
Supplement: Supplementary Figures S1-S7 and Table S1 [file BCJ-477-801-s1.pdf]

Supplementary Figure 1

a

GW301784X

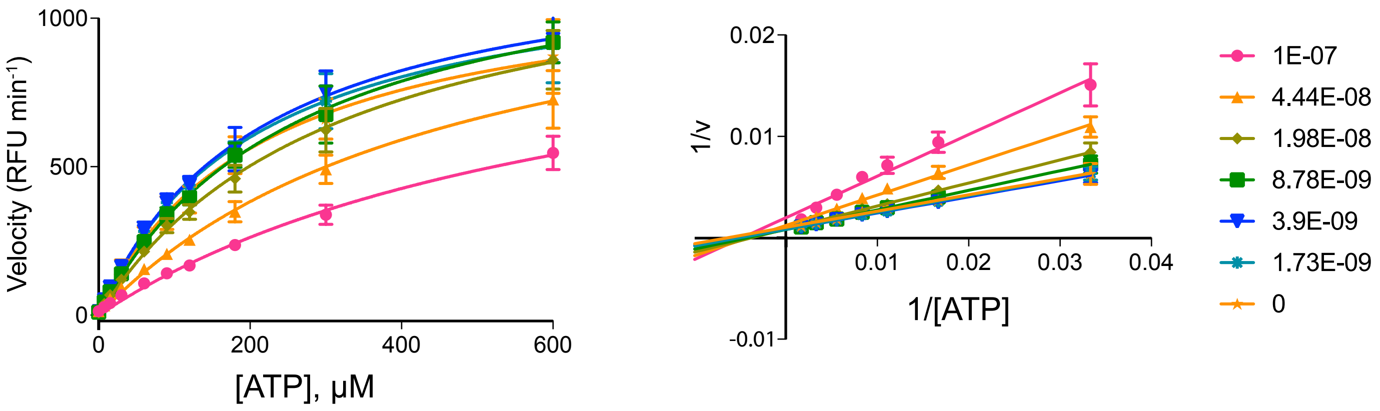

b

GW837331X

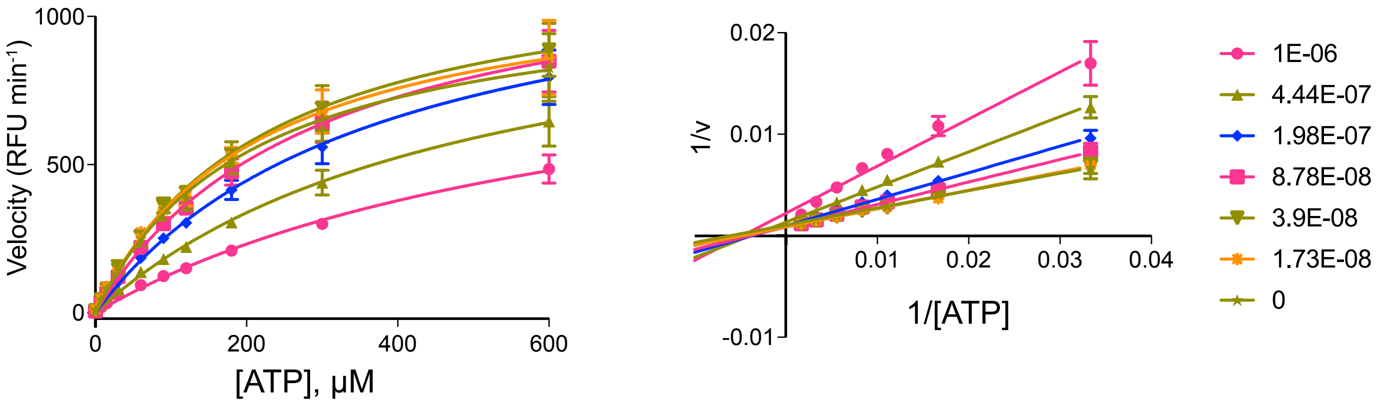

Supplementary figure 1: Michaelis-Menten and Lineweaver-Burke plots showing inhibition of ULK1 by (a) GW301784X and (b) GW837331X at varying concentrations of ATP. The data show the average of three independent experiments; error bars indicate the standard deviation.

Supplementary Figure 2

a

GW301784X

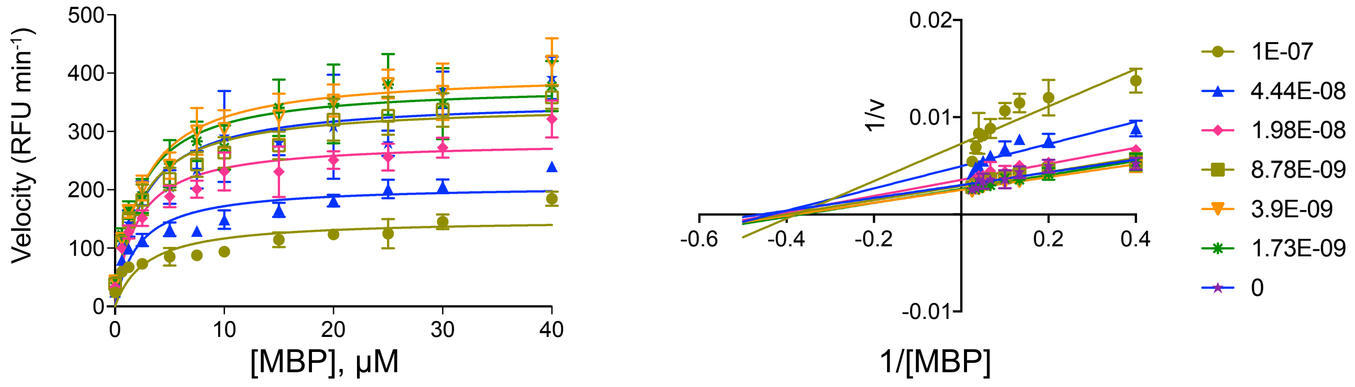

b

GW837331X

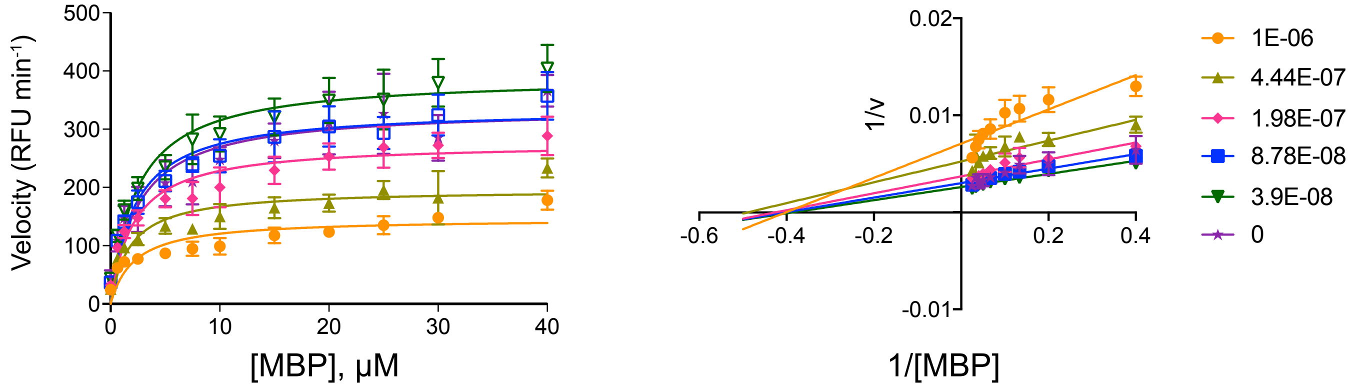

c

GW406108X

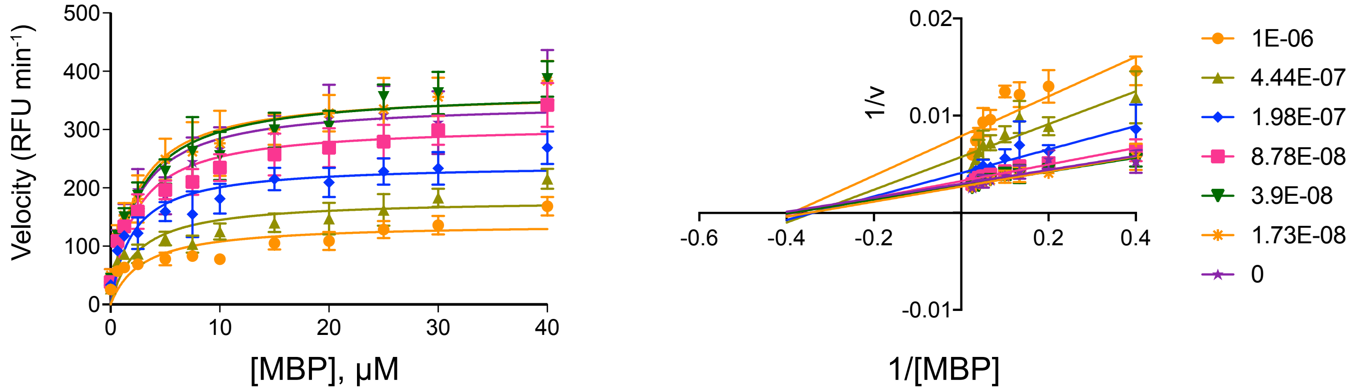

Supplementary figure 2: Michaelis-Menten and Lineweaver-Burke plots showing inhibition of ULK1 by (a) GW301784X, (b) GW837331X and (c) GW406108X at varying concentrations of MBP. The data show the average of three independent experiments; error bars indicate the standard deviation.

Supplementary Figure 3

a

GSK846226A

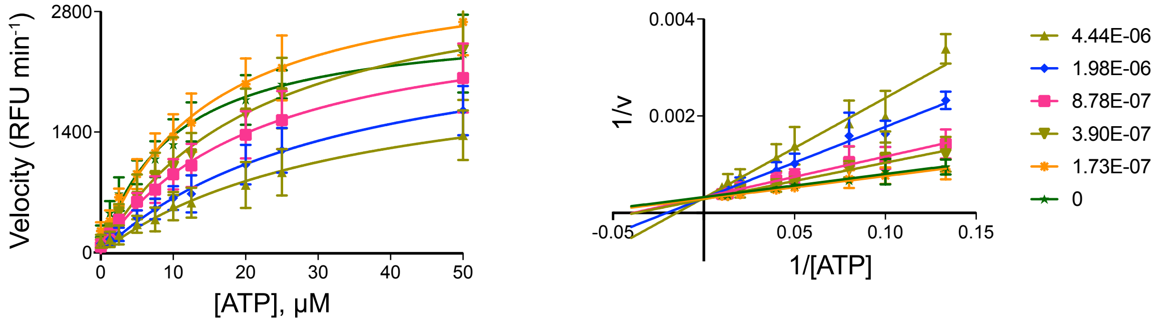

b

GW429374A

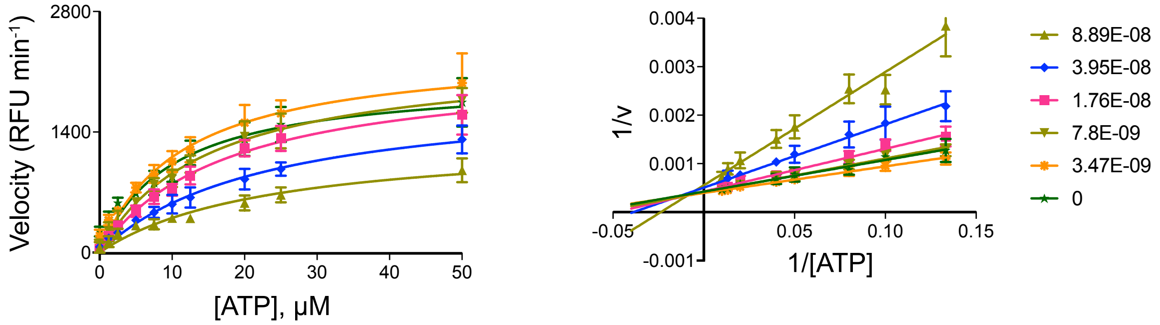

Supplementary figure 3: Michaelis-Menten and Lineweaver-Burke plots showing inhibition of VPS34 by (a) GSK846226A and (b) GW429374A at varying concentrations of ATP. The data show the average of three independent experiments; error bars indicate the standard deviation.

Supplementary Figure 4

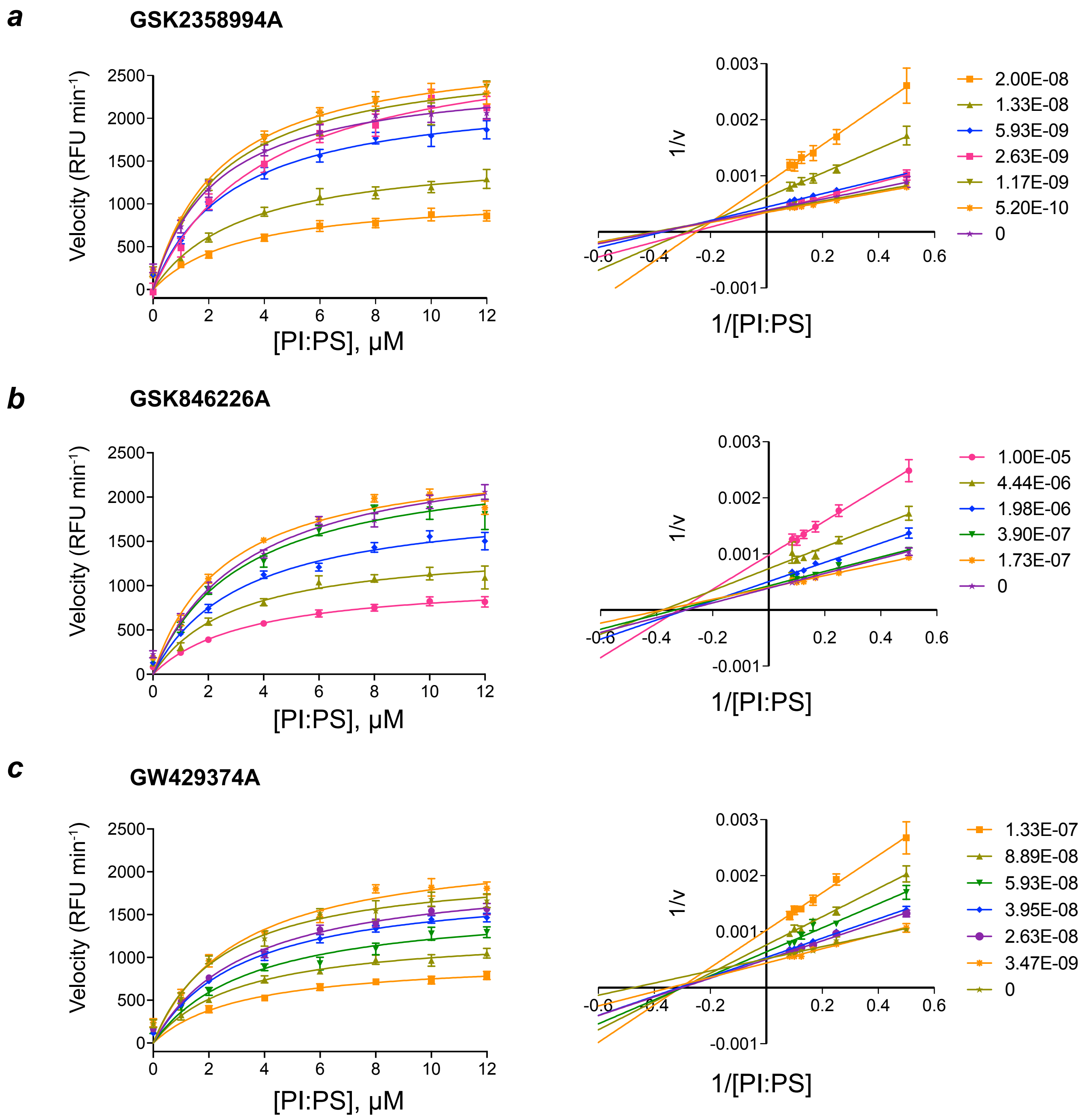

Supplementary figure 4: Michaelis-Menten and Lineweaver-Burke plots showing inhibition of VPS34 by (a) GSK2358994A, (b) GSK846226A and (c) GW429374A at varying concentrations of PI:PS. The data show the average of three independent experiments; error bars indicate the standard deviation.

Supplementary figure 5

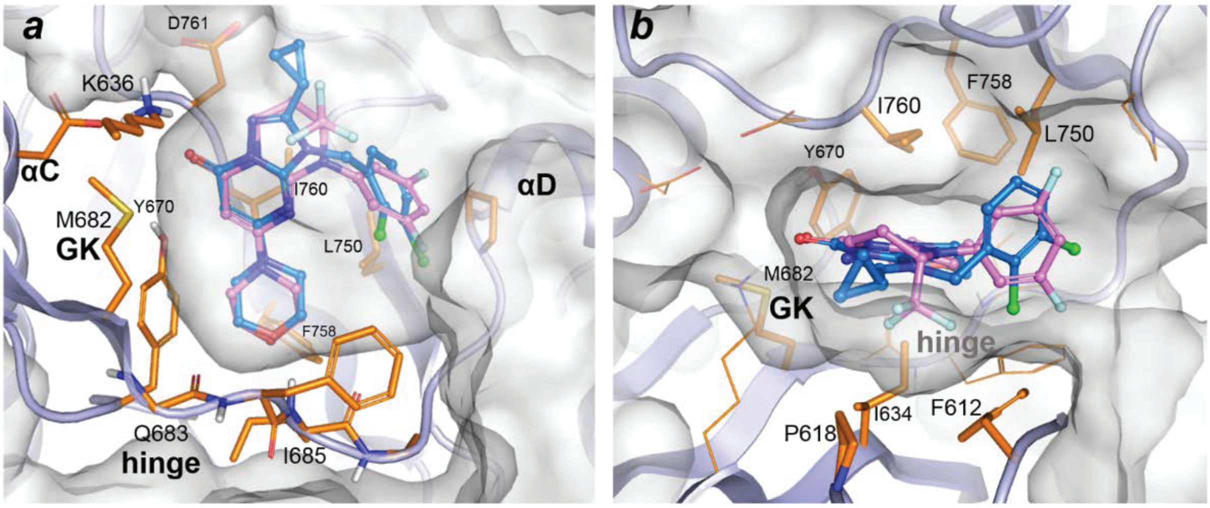

Supplementary figure 5: Superimposition of the docking pose of VPS34 inhibitor GSK2358994A (blue) and the X-ray structure of the morpholine analogue from 4UWF PDB entry (pink) in (a) lateral view and (b) top view.

Supplementary Figure 6

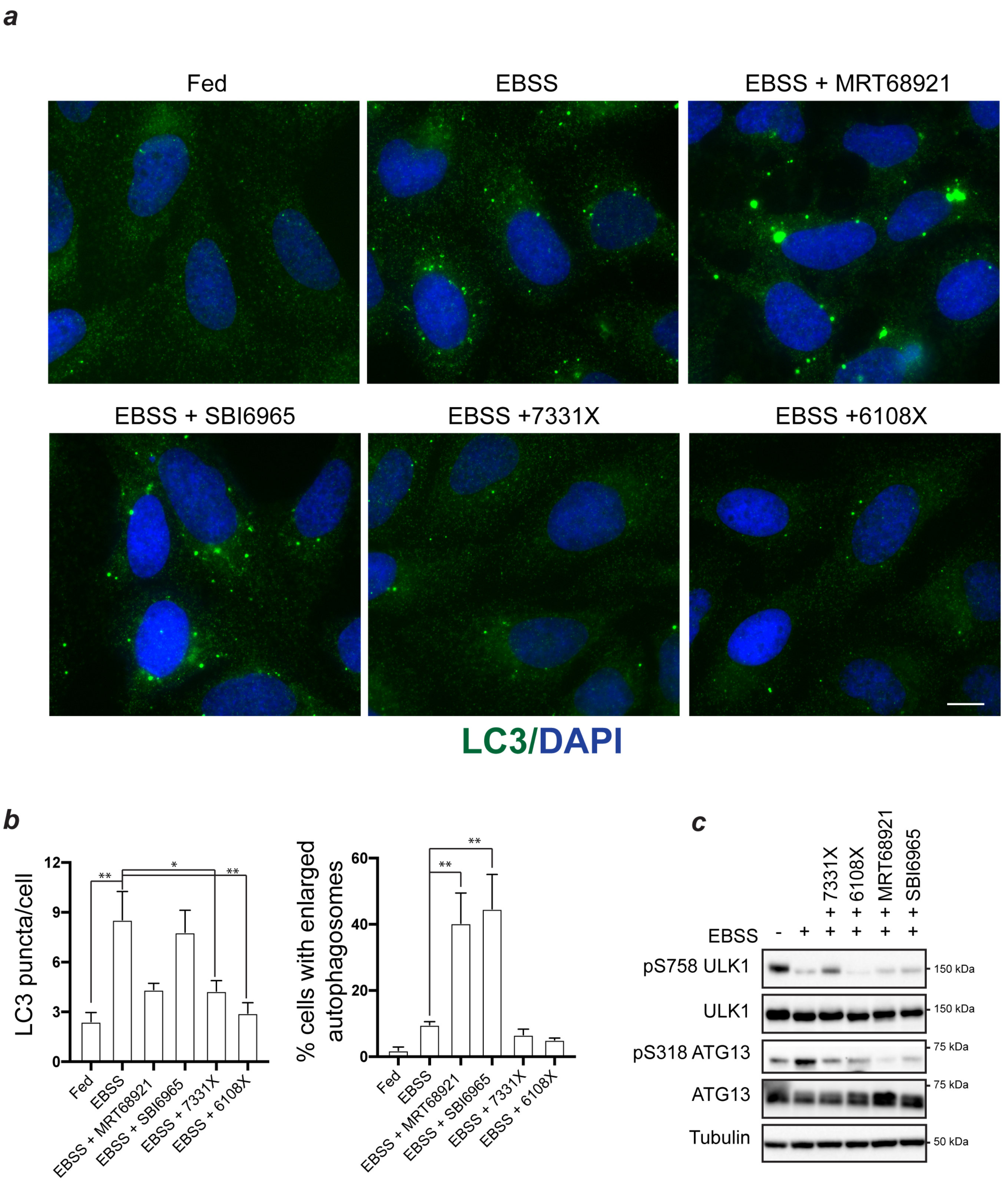

Supplementary figure 6: (a) U2OS cells were pre-treated for 60 min with 2 µM MRT68921 or 5 µM SBI0206965, 5 µM GW837331X or 5 µM GW406108X, following 120 min treatment with EBSS, as indicated. Cells were subsequently fixed and stained for endogenous LC3 (green) and DAPI (blue). Scale bar 10µm. (b) Quantification of LC3 puncta using Volocity and manual quantification of % of cells with enlarged LC3-stained autophagosomes. Graph bars represent mean of n=3 experiments ± SEM and statistics were carried out using one-way ANOVA and Dunnett's post-hoc test (graph representing number of LC3 puncta) or one-way ANOVA and Turkey's post-hoc test (graph representing % of cells with enlarged LC3-stained autophagosomes). * $p \leq 0.05$ and ** $p \leq 0.01$. (c) U2OS cells were treated as in (a). Cells were then subjected to western blot analysis with antibodies against the indicated proteins.

Supplementary Figure 7

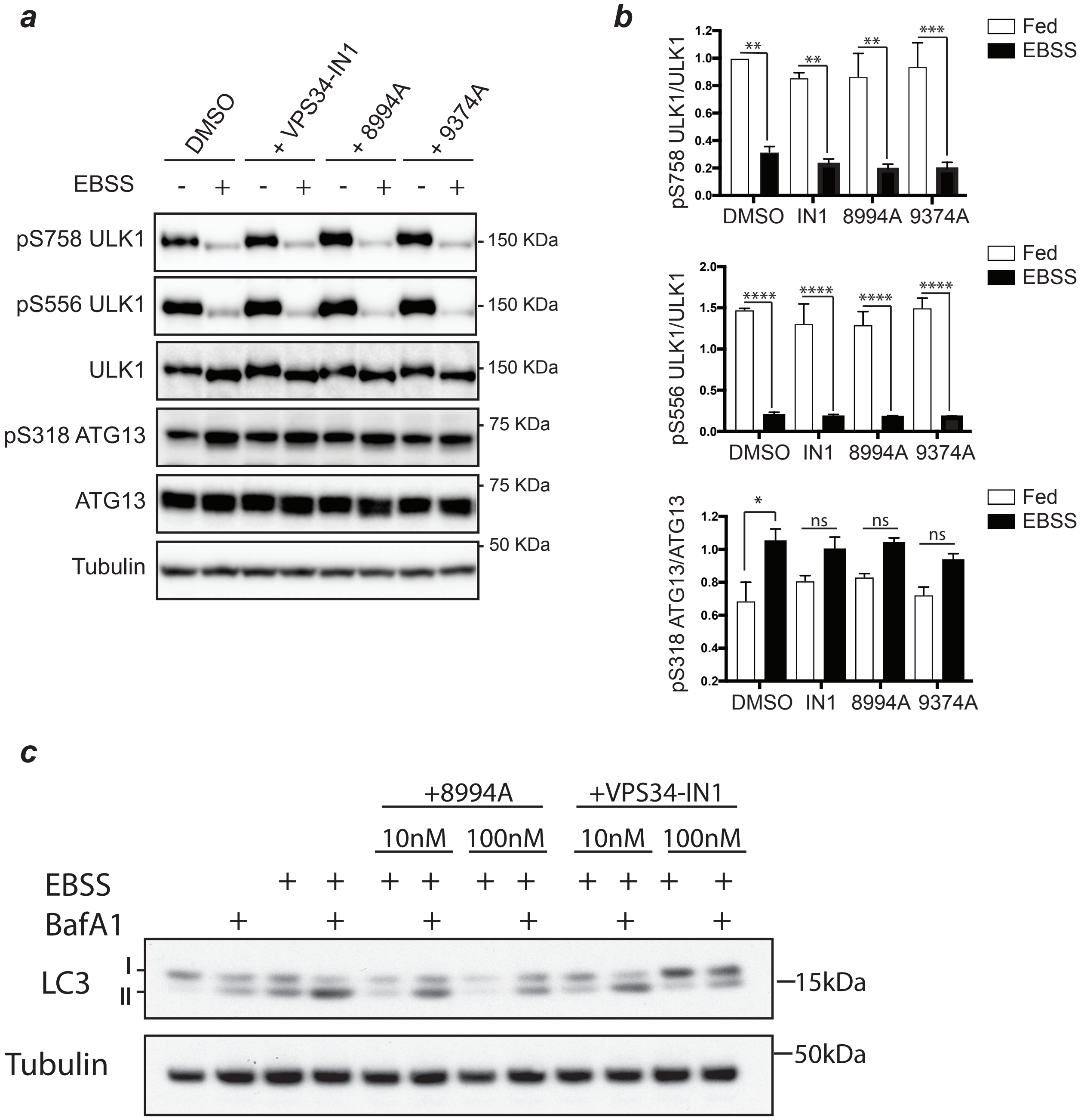

Supplementary figure 7: (a) U2OS cells were pre-treated for 60 min with 500 nM VPS34-IN1, 100 nM GSK2358994A or 100 nM GW429374A, following treatment with or without EBSS for 60 min, as indicated. Cells were subsequently subjected to western blot analysis with antibodies against the indicated proteins. (b) Quantification of pS758 ULK1 normalised to total ULK1 levels, pS556 ULK1 normalised to total ULK1 levels and pS318 ATG13 normalised to total ATG13 levels. Graph bars represent mean of n=3 experiments and statistics were carried out using one-way ANOVA and Turkey's post-hoc test. * $p \leq 0.05$, ** $p \leq 0.01$, *** $p \leq 0.001$, **** $p \leq 0.0001$ and ns = non-significant. (c) U2OS cells were pre-treated for 60 min with or without 10 nM or 100nM of GSK2358994A or VPS34-IN1 as indicated following 60 min treatment with or without EBSS and 50 nM BafA1. Cells were next subjected to western blot analysis with antibodies against LC3 and Tubulin.

Supplementary table 1

GW301784X	GW837331X	GW406108X	GSK2358994A	GW429374A	GSK846226A
CDK2	NEK9	CLK2	PKCd	IKKe	IKKb
CDK3	PLK1	TNK1		HGK	JAK2
MST1	FMS	DYRK1B		AurA	ROCK2
TTK	SRC	PKCb		CLK2	PKACa
MST2	MELK	DYRK1A		MINK	ROCK1
CDK5	EphB2	GSK3B		TNK1	TRKA
MELK	EphA4	HGK		PKCb	AurC
PDGFRb		FMS		DYRK1A	SRPK1
AMPKα1		GSK3A		GPRK7	DAPK1
GSK3B		PKCa		SRM	CLK3
GSK3A		AurA		PKCa	
TBK1		JAK3			
PDGFRa		MST1			
CLK2		MST2			
IKKe		CLK3			
AurA		KIT			
KDR		MINK			
CDK4		SRM			
FYN		RET			
MARK1		CK2a1			
BRSK2		GPRK7			
		HIPK1			
		PIM3			
		IKKe			

Supplementary table 1: List of kinases that are inhibited over 50% with each of the inhibitors shown (descending order). Kinase inhibition data extracted from the ChEMBL database (https://www.ebi.ac.uk/chembl/).
